# The role of noninvasive tests and liver biopsy in the diagnosis of nonalcoholic fatty liver disease

**DOI:** 10.25122/jml-2018-1002

**Published:** 2018

**Authors:** Craita Isabela Andronescu, Monica Roxana Purcarea, Petru Aurel Babes

**Affiliations:** 1. Medical Center Memormed, Bucharest; 2. Clinical Nephrology Hospital “Carol Davila”, Bucharest; 3. University of Oradea, Faculty of Medicine and Pharmacy

**Keywords:** nonalcoholic fatty liver disease, nonalcoholic steatohepatitis, liver biopsy, serological markers, elastography, CT, MRI

## Abstract

Nonalcoholic fatty liver disease (NAFLD) is characterized by hepatic steatosis in the absence of significant alcohol consumption (<40 g/week). The essential metabolic trait is insulin resistance, which is why NAFLD is associated with obesity, diabetes mellitus (DM), hyperlipidemia.

Approximately one-quarter of adults with NAFLD present nonalcoholic steatohepatitis (NASH) leading to progressive hepatic fibrosis and finally cirrhosis and hepatocellular carcinoma.

If liver biopsy (LB) has traditionally been NAFLD’s gold standard, over the past 15 years, its use has undergone an important transformation.

In this review, the role of noninvasive tests (serological markers, imaging techniques) in the NAFLD evaluation is analyzed, starting from the low adherence of patients for LB, the complications of the technique, and the increased cost.

LB is the only investigation that distinguishes between simple steatosis and NASH.

However, in the medical practice, LB has gained lesser value; it is worth mentioning that NASH represents a small proportion compared to NAFLD. For this reason, most patients only show biopsy steatosis, which has a good prognosis. In addition, judging by the appearance of inflammation markers and fibrosis in the diagnosis technique, the use of LB has become increasingly rare in the definition of NASH.

## Introduction

Nonalcoholic steatohepatitis was originally described by Ludwig in 1980 at the Mayo Clinic on a batch of patients who had DM and metabolic syndrome when alcohol consumption levels were insignificant.

Today, NASH is considered part of a spectrum of NAFLD [1]. NASH resembles alcoholic steatohepatitis but occurs in individuals who do not consume large amounts of alcohol (<40g/week).

Both the broader NAFLD entity and the NASH subtype are considered the hepatic manifestation of the metabolic syndrome [2]. Nonalcoholic steatohepatitis presents a risk of progressive liver fibrosis that can ultimately cause cirrhosis and hepatocellular carcinoma. If LB traditionally represented the gold standard for the diagnosis and prognosis of NAFLD, today its use in medical practice has undergone a change.

In the following paper, we will refer to the role of noninvasive tests (serologic markers, imaging techniques) taking into account the LB deficiencies (evaluation errors, complications, cost) and the lack of adherence of patients to this investigation.

## Liver biopsy (LB)

Liver biopsy is usually performed percutaneously. In some situations (severe obesity, thrombocytopenia, coagulation disorders), percutaneous LB is risk-ridden and can be performed by other routes (transjugular, laparoscopic, endoscopic).

The histological examination provides data on the existence and severity of steatosis, inflammation, and fibrosis. The fibrosis stage is established according to the system used in viral hepatitis F0 (without fibrosis) at F4 (cirrhosis) [3].

The sample harvested by LB may be the cause of interpretation errors. It is important to mention that the obtained liver fraction represents the 50.000^th^ part of the liver volume liver. In addition, the liver is not uniformly affected in NAFLD [4]. Ratziu et al. conducted a study on a group of 51 patients with NAFLD in whom two liver biopsies were performed the same day and reported that 35% of those with F3 in a sample presented F0 or F1 in the other sample [4].

The size of the fragment increases the precision of the diagnosis. Fragments larger than 3 cm bring high accuracy, but this is difficult to achieve in medical practice; most fragments do not exceed 1.5 cm. [5]. In the same study, Ratziu et al. found that the examination of a single biopsy sample showed a predictive negative value below 74% [4]. Another fact that reduces the accuracy of the diagnosis is the subjectivity of the interpretation, especially with respect to steatosis and inflammatory activity. Regarding the complications of the procedure, we mention post-procedural pain (50% of patients), hemorrhages, penetrating other organs (colon, kidneys), and death (0.1%). Finally, according to Tapper et al., the cost of the technique is high, involving a complex team of doctors. Performing LB on alternate paths raises the cost of the process even more.

## Noninvasive evaluation of fibrosis

Methods developed in recent years for a noninvasive assessment of fibrosis can distinguish between early and advanced fibrosis but cannot appreciate its severity. [6] A. Serological markers (indirect or direct). 1. Indirect markers a) AST and ALT levels may be elevated without exceeding 2 times the ULN (upper limit of normal). The AST/ALT ratio is increased by decreasing AST clearance and decreasing ALT production when cirrhosis occurs. [7] b) The serum alkaline phosphatase level is slightly increased in nearly 50% of patients. c) Approximately 10 to 15% of patients experience indirect hyperbilirubinemia. d) Liver synthesis tests (prothrombin time and serum albumin level) are normal if cirrhosis has not occurred. e) Thrombocytopenia occurs by splenic sequestration but also by decreasing thrombopoietin production [8,9]. This is the earliest indicator of severe fibrosis and it is the basis for calculating risk scores for fibrosis, along with the value of aminotransferases [8] and some proteins that are deficient in hepatic failure (coagulation factors, haptoglobin, apolipoprotein A). [10] The NAFLD score also includes BMI and markers for DM (HbA1c, fasting glycaemia, impaired fasting glucose – IFG) [11].

These scores are: • The AST/platelet index is calculated as follows: (AST ÷ ULN) ÷ no of platelets • The Fibrosis-4 score (Fib4) is calculated according to the formula: (age x AST): no. of platelets x √ALT • The NAFLD fibrosis score (11) is calculated using the formula: • 1.675 + 0.037 x age + 0.094 x BMI + 1.13 x RI (resistance insulin) or diabetes (yes = 1, no = 0) + 0.99 x AST ratio: ALT – 0.013 x no. of platelets – 0.66 x albumin • The LOK index (by decreasing the coagulation factors in the cirrhosis) it is calculated by the log-odds formula = – 5.56 – 0.0089 x no. of platelets + 1.26 x AST / ALT +5.27 x INR These scores cannot be routinely used because of the high cost. Sometimes, difficulties with differential diagnosis may be encountered; e.g., thrombocytopenia may be owed to hematological causes, while alcohol and drug abuse, concomitant muscle disease may lead to increased serum aminotransferases. As there are 2 cutoff values to maximize specificity and sensitivity that create undetermined areas (neither high risk nor low risk), LB is required in this situation for the evaluation of the fibrosis stage (F1 – F4). 2. Direct markers They are represented by proteins that assess both fibrogenesis and fibrinolysis (<#125>2 macroglobulin, hyaluronic acid, N-terminal propeptide type III procollagen type, metalloproteinase tissue inhibitor). These markers are commercially available under various names (Fibrotest, Fibrospect, Enhanced Liver Fibrosis Score). B. Imaging tests They do not distinguish simple steatosis from NASH; NASH is usually diffused while steatosis can be diffused or focal. a) Ultrasonography The liver appears hyperechogenic or “bright”. Steatosis is especially detected when fat accumulates excessively. In cirrhosis, the liver is echogenic, with a thicker texture and it is nodular with an enlarged caudate lobe. Changed hepatic contour has no diagnostic value [12]. Diagnosis of cirrhosis is confirmed by splenomegaly, an increase in portal vein diameter, ascites, venous abdominal circulation. b) CT In hepatic steatosis, the liver appears hypodense compared to the spleen on native CT. c) MRI MRI is the most sensitive noninvasive test for fatty liver detection. In the case of focal steatosis, a signal intensity loss on T1-weighted images is noticed. On CT and MRI, focal steatosis is visible peripherally (in insulin-dependent diabetics treated by peritoneal dialysis), centrally, and periportally; it does not present in geometric shapes (such as spherical) and does not compress the adjacent structures. Differential diagnosis is made with hemangioma that is hyperechogenic, but slightly hypoechoic in the liver. Dilated intrahepatic bile ducts are hardly visible in the fatty liver. CT and MRI are superior to ultrasound scans in NAFLD evaluation. d) Elastography Elastography is more accurate than other imaging techniques. There are several techniques, the most commonly used being transient elastography and MRI elastography [13]. It is more useful to exclude fibrosis than to confirm it. It should be mentioned that increased hepatic stiffness is also encountered in stasis liver, severe inflammation, and so forth. Errors of appreciation are higher in obese patients; in these situations, MRI elastography is preferred [13,14]. If none of the elastographic methods are conclusive, LB can be performed. Risk stratification in both advanced fibrosis and moderate fibrosis has largely replaced the anatomopathological staging from F0→F4. This stratification allows a better discrimination between different fibrosis stages (small, moderate or high). The strategy of using biopsy only in the case of undetermined probability reduces the number of biopsies by 70% in those undergoing this risk analysis, compared to those on whom LB is performed from the beginning. Based on FIB-4 and NAFLD-Fibrosis scores, researchers like Shah et al. or Angulo et al., showed that 28% of patients had an undetermined risk. [15,16] Hence, it is useful to perform two tests with concurrent results (one serological and one imagistic or elastographic). These patients can be divided into one of three risk factors: concordantly reduced, concordantly raised, discordant or undetermined. Approximately 21% of the patients will experience undetermined results. These tests can be repeated on another medical visit. Using this consistent strategy, tests are performed on 70% of those with discordant results, eliminating the need for biopsy. Because serologic tests (Fib-4, NAFLD-Fibrosis) identify patients with advanced fibrosis and undetermined scores, the primary care doctor may refer them to a hepatologist. The hepatologist can make therapeutic decisions in advanced fibrosis when the area under the ROC curve (area under ROC) is ≥ 0.08; nowadays, early complications of HTP, liver cancer, can be detected. From an elastographic point of view, cirrhosis accompanies a liver rigidity of 12.5 kPa. The prognosis is all the more severe as the stiffness is higher (> 20 kPa, even up to 50 kPa). In 2015, the Baveno VI Consensus Workshop found that patients with a platelet count of >150,000/mm^3^ and hepatic stiffness <20 KPa do not require esophageal varices screening, as there is no risk of complications. [17].

## The practical utility of noninvasive tests

The diagnosis of NAFLD is usually reached by measuring the serum elevation of liver enzymes. Other diseases that cause an increase in serum liver enzymes are alcoholic liver disease (ALD) and viral hepatitis.

The clinical examination in conjunction with serological markers and imaging is sufficient for diagnosis. Anamnesis plays a role in the diagnosis of alcoholic liver disease (ALD) and imaging for hepatic steatosis. Serological tests have no role in distinguishing between these diseases.

Several studies coordinated by different authors showed that after the initial evaluation, 77% of the undiagnosed patients who were evaluated for LB had NAFLD and ALD. [18-20]

In those suspected of NAFLD with metabolic syndrome or obesity and inconclusive ultrasound, elastography is recommended by both techniques mentioned above.

Noninvasive tests do not exhibit LB complications, are less costly, and can be repeated in evolving monitoring.

LB continues to play a role in NAFLD management, differentiating simple steatosis from NASH. Although NASH circulating biomarkers such as cytokeratin 18 and plasminogen activator inhibitor-1 seem promising in this regard, they are not approved for medical practice. The FDA in the US has not validated any medication because it claims a histological proof of improvement, which involves at least two biopsies performed in clinical trials (something patients find difficult to accept).

## Conclusions

Noninvasive tests (serological and imaging techniques) in conjunction with the clinical examination are sufficient in most cases for the diagnosis of NAFLD.

In patients suspected with NAFLD due to obesity and metabolic syndrome, transient elastography and MRI elastography can confirm the presence of hepatic steatosis. LB continues to play a role in the treatment of NAFLD, although severity may be over- or underestimated by histopathological examination, by interobserver variation.

## Conflict of Interest

The authors confirm that there are no conflicts of interest.
